# Supplementary material for: Unveiling the Bioactive Potential of the Invasive Jellyfish Phyllorhiza punctata Through Integrative Transcriptomic and Proteomic Analyses
Source: Biomolecules. 2025 Aug 4;15(8):1121. doi: 10.3390/biom15081121 (PMC12383608; doi:10.3390/biom15081121)
Supplement: Supplementary file 1 [file biomolecules-15-01121-s001.zip › Suplementary Material/FileS3/PEPFOLD4/XP_065058870_7165/clusters.html]

 PEP-FOLD clusters


Download archive of all models  here   

|  |  |  |
| --- | --- | --- |
| # | file | sOPEP |
| # cluster 1 - Size: 5 |  |  |
|  | XP\_065058870\_7165-model1 | -27.6127 |
|  | XP\_065058870\_7165-model1.1 | -25.9686 |
|  | XP\_065058870\_7165-model1.2 | -24.9182 |
|  | XP\_065058870\_7165-model1.3 | -24.342 |
|  | XP\_065058870\_7165-model1.4 | -23.4955 |
| # cluster 2 - Size: 3 |  |  |
|  | XP\_065058870\_7165-model2 | -27.4634 |
|  | XP\_065058870\_7165-model2.1 | -24.7806 |
|  | XP\_065058870\_7165-model2.2 | -24.3964 |
| # cluster 3 - Size: 5 |  |  |
|  | XP\_065058870\_7165-model3 | -27.1226 |
|  | XP\_065058870\_7165-model3.1 | -26.9869 |
|  | XP\_065058870\_7165-model3.2 | -26.7782 |
|  | XP\_065058870\_7165-model3.3 | -26.6104 |
|  | XP\_065058870\_7165-model3.4 | -24.6058 |
| # cluster 4 - Size: 1 |  |  |
|  | XP\_065058870\_7165-model4 | -26.8494 |
| # cluster 5 - Size: 4 |  |  |
|  | XP\_065058870\_7165-model5 | -26.3709 |
|  | XP\_065058870\_7165-model5.1 | -26.115 |
|  | XP\_065058870\_7165-model5.2 | -25.5193 |
|  | XP\_065058870\_7165-model5.3 | -23.3345 |
| # cluster 6 - Size: 1 |  |  |
|  | XP\_065058870\_7165-model6 | -26.3615 |
| # cluster 7 - Size: 6 |  |  |
|  | XP\_065058870\_7165-model7 | -26.3241 |
|  | XP\_065058870\_7165-model7.1 | -25.9006 |
|  | XP\_065058870\_7165-model7.2 | -25.3446 |
|  | XP\_065058870\_7165-model7.3 | -25.2713 |
|  | XP\_065058870\_7165-model7.4 | -24.5383 |
|  | XP\_065058870\_7165-model7.5 | -24.4049 |
| # cluster 8 - Size: 2 |  |  |
|  | XP\_065058870\_7165-model8 | -26.3202 |
|  | XP\_065058870\_7165-model8.1 | -24.5344 |
| # cluster 9 - Size: 1 |  |  |
|  | XP\_065058870\_7165-model9 | -26.0378 |
| # cluster 10 - Size: 4 |  |  |
|  | XP\_065058870\_7165-model10 | -25.9166 |
|  | XP\_065058870\_7165-model10.1 | -25.5901 |
|  | XP\_065058870\_7165-model10.2 | -24.5698 |
|  | XP\_065058870\_7165-model10.3 | -24.4494 |
| # cluster 11 - Size: 7 |  |  |
|  | XP\_065058870\_7165-model11 | -25.8757 |
|  | XP\_065058870\_7165-model11.1 | -25.6932 |
|  | XP\_065058870\_7165-model11.2 | -25.6033 |
|  | XP\_065058870\_7165-model11.3 | -25.1608 |
|  | XP\_065058870\_7165-model11.4 | -25.0998 |
|  | XP\_065058870\_7165-model11.5 | -24.217 |
|  | XP\_065058870\_7165-model11.6 | -23.9394 |
| # cluster 12 - Size: 1 |  |  |
|  | XP\_065058870\_7165-model12 | -25.8479 |
| # cluster 13 - Size: 2 |  |  |
|  | XP\_065058870\_7165-model13 | -25.7209 |
|  | XP\_065058870\_7165-model13.1 | -23.7903 |
| # cluster 14 - Size: 2 |  |  |
|  | XP\_065058870\_7165-model14 | -25.3394 |
|  | XP\_065058870\_7165-model14.1 | -25.2208 |
| # cluster 15 - Size: 2 |  |  |
|  | XP\_065058870\_7165-model15 | -25.3098 |
|  | XP\_065058870\_7165-model15.1 | -24.3722 |
| # cluster 16 - Size: 2 |  |  |
|  | XP\_065058870\_7165-model16 | -25.2913 |
|  | XP\_065058870\_7165-model16.1 | -24.0492 |
| # cluster 17 - Size: 2 |  |  |
|  | XP\_065058870\_7165-model17 | -25.2747 |
|  | XP\_065058870\_7165-model17.1 | -24.3013 |
| # cluster 18 - Size: 1 |  |  |
|  | XP\_065058870\_7165-model18 | -25.2581 |
| # cluster 19 - Size: 2 |  |  |
|  | XP\_065058870\_7165-model19 | -25.0975 |
|  | XP\_065058870\_7165-model19.1 | -24.8054 |
| # cluster 20 - Size: 2 |  |  |
|  | XP\_065058870\_7165-model20 | -25.0829 |
|  | XP\_065058870\_7165-model20.1 | -24.3886 |
| # cluster 21 - Size: 5 |  |  |
|  | XP\_065058870\_7165-model21 | -24.8749 |
|  | XP\_065058870\_7165-model21.1 | -24.6383 |
|  | XP\_065058870\_7165-model21.2 | -24.3597 |
|  | XP\_065058870\_7165-model21.3 | -24.235 |
|  | XP\_065058870\_7165-model21.4 | -23.7687 |
| # cluster 22 - Size: 4 |  |  |
|  | XP\_065058870\_7165-model22 | -24.8328 |
|  | XP\_065058870\_7165-model22.1 | -24.8169 |
|  | XP\_065058870\_7165-model22.2 | -24.38 |
|  | XP\_065058870\_7165-model22.3 | -24.1935 |
| # cluster 23 - Size: 4 |  |  |
|  | XP\_065058870\_7165-model23 | -24.8119 |
|  | XP\_065058870\_7165-model23.1 | -24.7724 |
|  | XP\_065058870\_7165-model23.2 | -24.4539 |
|  | XP\_065058870\_7165-model23.3 | -24.3934 |
| # cluster 24 - Size: 1 |  |  |
|  | XP\_065058870\_7165-model24 | -24.6539 |
| # cluster 25 - Size: 3 |  |  |
|  | XP\_065058870\_7165-model25 | -24.5623 |
|  | XP\_065058870\_7165-model25.1 | -24.3334 |
|  | XP\_065058870\_7165-model25.2 | -23.5943 |
| # cluster 26 - Size: 1 |  |  |
|  | XP\_065058870\_7165-model26 | -24.5365 |
| # cluster 27 - Size: 2 |  |  |
|  | XP\_065058870\_7165-model27 | -24.4417 |
|  | XP\_065058870\_7165-model27.1 | -23.7882 |
| # cluster 28 - Size: 1 |  |  |
|  | XP\_065058870\_7165-model28 | -24.35 |
| # cluster 29 - Size: 1 |  |  |
|  | XP\_065058870\_7165-model29 | -24.3165 |
| # cluster 30 - Size: 1 |  |  |
|  | XP\_065058870\_7165-model30 | -24.2612 |
| # cluster 31 - Size: 1 |  |  |
|  | XP\_065058870\_7165-model31 | -24.1159 |
| # cluster 32 - Size: 2 |  |  |
|  | XP\_065058870\_7165-model32 | -24.1051 |
|  | XP\_065058870\_7165-model32.1 | -23.3633 |
| # cluster 33 - Size: 2 |  |  |
|  | XP\_065058870\_7165-model33 | -24.065 |
|  | XP\_065058870\_7165-model33.1 | -22.9919 |
| # cluster 34 - Size: 1 |  |  |
|  | XP\_065058870\_7165-model34 | -24.0424 |
| # cluster 35 - Size: 1 |  |  |
|  | XP\_065058870\_7165-model35 | -24.0415 |
| # cluster 36 - Size: 1 |  |  |
|  | XP\_065058870\_7165-model36 | -23.9289 |
| # cluster 37 - Size: 1 |  |  |
|  | XP\_065058870\_7165-model37 | -23.8314 |
| # cluster 38 - Size: 1 |  |  |
|  | XP\_065058870\_7165-model38 | -23.6694 |
| # cluster 39 - Size: 1 |  |  |
|  | XP\_065058870\_7165-model39 | -23.571 |
| # cluster 40 - Size: 5 |  |  |
|  | XP\_065058870\_7165-model40 | -23.5344 |
|  | XP\_065058870\_7165-model40.1 | -23.519 |
|  | XP\_065058870\_7165-model40.2 | -23.261 |
|  | XP\_065058870\_7165-model40.3 | -23.1116 |
|  | XP\_065058870\_7165-model40.4 | -22.5591 |
| # cluster 41 - Size: 1 |  |  |
|  | XP\_065058870\_7165-model41 | -23.4004 |
| # cluster 42 - Size: 1 |  |  |
|  | XP\_065058870\_7165-model42 | -23.2117 |
| # cluster 43 - Size: 1 |  |  |
|  | XP\_065058870\_7165-model43 | -23.1394 |
| # cluster 44 - Size: 1 |  |  |
|  | XP\_065058870\_7165-model44 | -23.097 |
| # cluster 45 - Size: 1 |  |  |
|  | XP\_065058870\_7165-model45 | -22.7939 |
| # cluster 46 - Size: 1 |  |  |
|  | XP\_065058870\_7165-model46 | -22.7538 |
|  |  |  |
